# Spatial and Temporal Distribution of *Culicoides* Species in Mainland Portugal (2005–2010). Results of the Portuguese Entomological Surveillance Programme

**DOI:** 10.1371/journal.pone.0124019

**Published:** 2015-04-23

**Authors:** Rita Ribeiro, Anthony J. Wilson, Telmo Nunes, David W. Ramilo, Rita Amador, Sara Madeira, Filipa M. Baptista, Lara E. Harrup, Javier Lucientes, Fernando Boinas

**Affiliations:** 1 Centre for Interdisciplinary Research in Animal Health (CIISA), Faculty of Veterinary Medicine, University of Lisbon, Lisbon, Portugal; 2 Integrative Entomology Group, The Pirbright Institute, Pirbright, Woking, Surrey, United Kingdom; 3 Direção-Geral de Alimentação e Veterinária, Food and Veterinary Central Services, Campo Grande, Lisbon, Portugal; 4 Entomology Group, Vector-borne Viral Diseases Programme, The Pirbright Institute, Ash Road, Pirbright, Woking, Surrey, United Kingdom; 5 Parasitology and Parasitic Diseases, Department of Animal Pathology (Animal Health), Veterinary Faculty, University of Zaragoza, Zaragoza, Spain; Institut Pasteur, FRANCE

## Abstract

Bluetongue virus (BTV) is transmitted by *Culicoides* biting midges and causes an infectious, non-contagious disease of ruminants. It has been rapidly emerging in southern Europe since 1998. In mainland Portugal, strains of BTV belonging to three serotypes have been detected: BTV-10 (1956-1960), BTV-4 (2004-2006 and 2013) and BTV-1 (2007-2012). This paper describes the design, implementation and results of the Entomological Surveillance Programme covering mainland Portugal, between 2005 and 2010, including 5,650 caches. *Culicoides imicola* Kieffer was mostly found in central and southern regions of Portugal, although it was sporadically detected in northern latitudes. Its peak activity occurred in the autumn and it was active during the winter months in limited areas of the country. Obsoletus group was present at the highest densities in the north although they were found throughout the country in substantial numbers. *Culicoides* activity occurred all year round but peaked in the spring. A generalized linear mixed model was developed for the analysis of the environmental factors associated with activity of the species of *Culicoides* suspected vectors of BTV in the country. For *C*. *imicola* Kieffer, the most important variables were month, diurnal temperature range (DTR), the number of frost days (FRS) and median monthly temperature (TMP). For the Obsoletus group, the most important factors were month, diurnal temperature range (DTR), and linear and quadratic terms for median monthly temperature (TMP). The results reported can improve our understanding of climatic factors in *Culicoides* activity influencing their distribution and seasonal pattern.

## Introduction

Bluetongue (BT) is an infectious disease of ruminants caused by bluetongue virus (BTV), an arbovirus with 26 recognised serotypes [1]. Clinical disease is most common in sheep and certain species of deer [2] but is also seen in cattle [3,4]. Outbreaks of BT often have a high economic impact, partly as a consequence of the restrictions on domestic and international animal movement following confirmation of an outbreak. Since 1998, BTV activity in Europe and in Mediterranean basin has increased substantially [5] and since 2006 the virus has emerged in northern Europe.

Bluetongue virus is transmitted primarily by several species of *Culicoides* (Diptera: Ceratopogonidae). As a consequence, the distribution and intensity of BTV infection are dependent on the distribution and abundance of the *Culicoides* vectors [6]. Although the Afrotropical species *C*. *imicola* Kieffer is considered to be the most important vector in most parts of Africa and southern Europe, species in the Obsoletus group and group Pulicaris have also been implicated as efficient vectors of certain strains of BTV during recent outbreaks in northern Europe [2,7]. In addition, vertical transmission in cattle has been observed for at least one field strain of BTV [8,9] and oral transmission is suspected [10], possibly explaining the observed ability of BTV to “overwinter” for long periods in the absence of vector activity [11].

**Fig 1:**
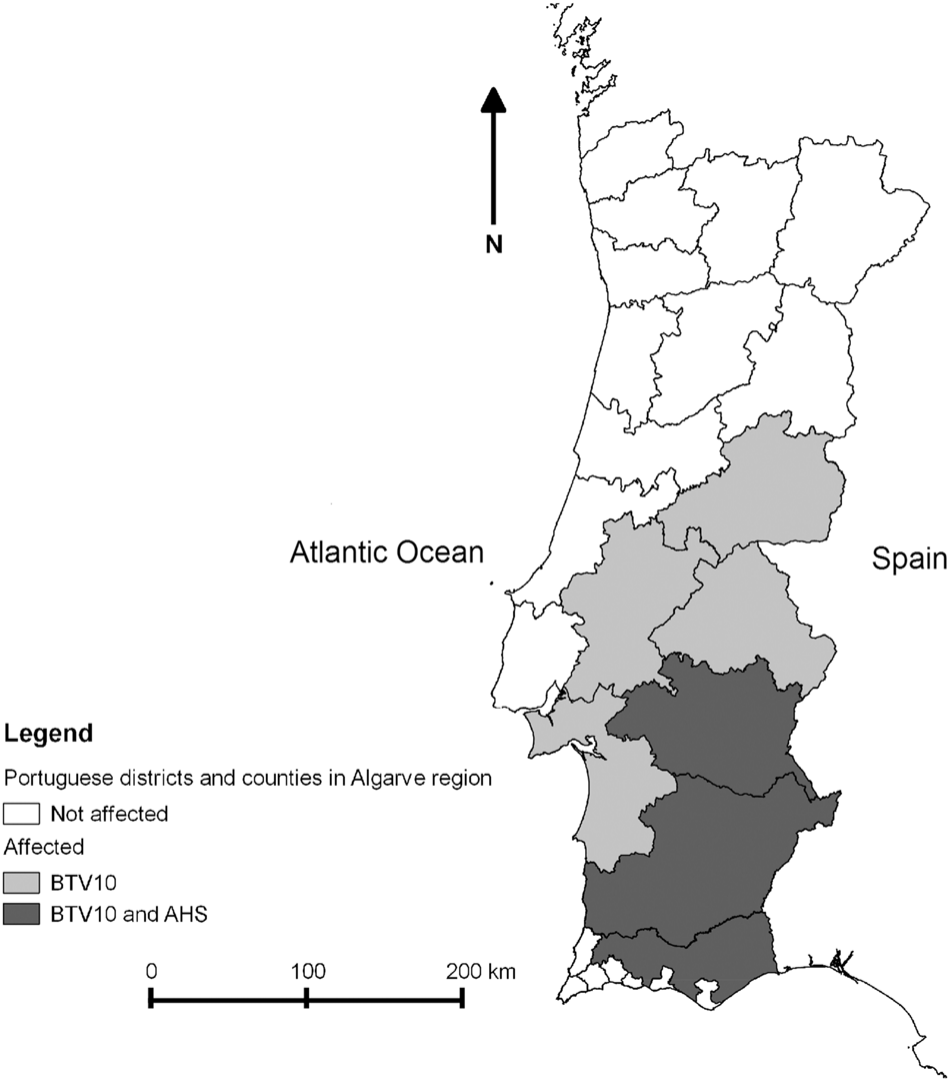
Historical extent of bluetongue virus and African horse sickness virus outbreaks in Portugal before 2004.

Bluetongue virus and other Culicoides-borne viruses have caused a number of outbreaks in Portugal since the middle of the twentieth century. The first recorded outbreak of BTV in Portugal began in 1956 and affected the southern and central eastern regions of the country (Fig 1). This outbreak was caused by a strain of BTV-10 which was probably introduced by windborne *Culicoides* from Morocco [12]. A monovalent live attenuated vaccine supplied by the Onderstepoort Veterinary Institute in South Africa was used to vaccinate sheep in the affected area of the country and the outbreak was largely eradicated by 1958, although the region was not declared free of disease until 1960 [13]. Another *Culicoides*-borne virus, African horse sickness virus (AHSV), was then introduced in 1989, most probably via windborne *Culicoides* blown across the Guadiana River with origin in the 1987–1990 Spanish epizootic [14]. The resulting outbreak was restricted to the southern region (Fig 1) of Portugal and was eradicated after massive vaccination of the *equidae* species in the whole country using a live attenuated monovalent vaccine again provided by the Onderstepoort laboratory in South Africa [14]. A second strain of BTV (this time of BTV-4) appeared in November 2004 in areas of the central-west and southern regions of Portugal bordering Spain. This resulted in an outbreak which persisted until the end of 2006 [13]. In 2007 a strain of BTV-1 was detected in the Alentejo region, in September 2007, in a county located at the Spanish border, two months after a BTV-1 outbreak began in Spain [15]. Between September and December 2007, 158 BTV-1 outbreaks were reported in regions in the south and center of country. However, since 2008, BTV-1 has spread to the north of the country, showing a different distribution than BTV-4, BTV-10 and AHSV. In 2008, 78 BTV-1outbreaks were reported in the center and south (83%) and in the northern (17%) regions. In 2009 the majority of the 129 BTV-1outbreaks were reported in the northern regions (67%) and in 2010 only six BTV-1 outbreaks occurred in the center and southern regions.

This BTV-1strain may still be circulating in the area, although at very low levels; only one outbreak was confirmed in 2011 and other three outbreaks in 2012.

In 2013 BTV-4 re-emerged again in the mainland territory, causing 10 outbreaks in Algarve region [15–17]. The potential exists for other disease-causing Culicoides-borne viruses, such as the recently-identified Schmallenberg virus (SBV) [18], to be introduced to Portugal via windborne vector transportation or the accidental importation of infected hosts potentially with substantial consequences for animal health and welfare, international trade and livestock production.

In recognition of the high and continuing threat to the Portuguese livestock sector from *Culicoides*-borne viruses, the Portuguese authorities established a National Entomological Surveillance Programme (ESP) in 2005, with the objective of collecting and identifying *Culicoides* species in Portugal, mapping their distribution and quantifying their abundance and association with outbreaks of *Culicoides*-transmitted diseases. The ESP was reduced in scope in 2010 because BTV activity in Portugal had declined since 2010. The archipelagos of Azores and Madeira were also included in the programme, but as BTV was absent from these regions, the programme in the islands was carried out with a different design using less frequent sampling.

As a consequence of the continuing emergence of BTV in Europe since 1998, similar national entomological surveillance programmes have been established in several affected countries.

The data generated by such programmes is potentially very valuable for a variety of purposes such as identifying suitable habitats for vectors, describing vectors distribution, continental-level transmission modelling and the development of vector population models that are capable of describing the response of vector populations to a broad range of environmental conditions, potentially allowing the consequences of future environmental change to be anticipated.

Only one work with the aim to predict the presence and abundance of *C*. *imicola* in Portugal has been published. Tatem *et al*., 2003 developed a set of models using trapping data obtained between 2000 and 2001, and described by Capela *et al*., (2003), together with 41 remotely-sensed variables [19]. Four variables were considered to have environmental significance: normalized difference vegetation index (NDVI, a measure correlated with soil moisture, rainfall and vegetation biomass, coverage and productivity), middle infra-red reflectance (MIR, correlated with the water content, surface temperature and structure of vegetation canopies), land surface temperature (LST) and air temperature (TAIR) [19]. This study has shown that what determines the presence of *C*. imicola is strongly correlated with middle infra-red reflectance, and consequently also correlated with vegetation structure and surface temperature, and how much each varies throughout the year. The abundance of this species was determined by the annual timing of the vegetation abundance peak, itself related to soil moisture levels [19].

However, this study does not evaluate determinants for Obsoletus group presence and abundance. Since BTV outbreaks in northern Europe specimens belonging to this group, are recognized as important vectors of disease [7].

In this manuscript, we describe the findings of the official Entomological Surveillance Programme from mainland Portugal during the period from September 2005 to August 2010, when the national entomological surveillance programme was fully in place and the majority of cases of BTV were detected. We also use a generalised linear mixed model (GLMM) to help understand the ability of climatic drivers commonly cited as important for vector activity to explain the probability of detecting *C*. *imicola* or Obsoletus group during our collections, and compare our insights into the importance of climatic drivers to those suggested by similar analyses of *Culicoides* activity, distribution or abundance in Portugal.

## Materials and Methods

### Entomological Surveillance Programme

**Fig 2:**
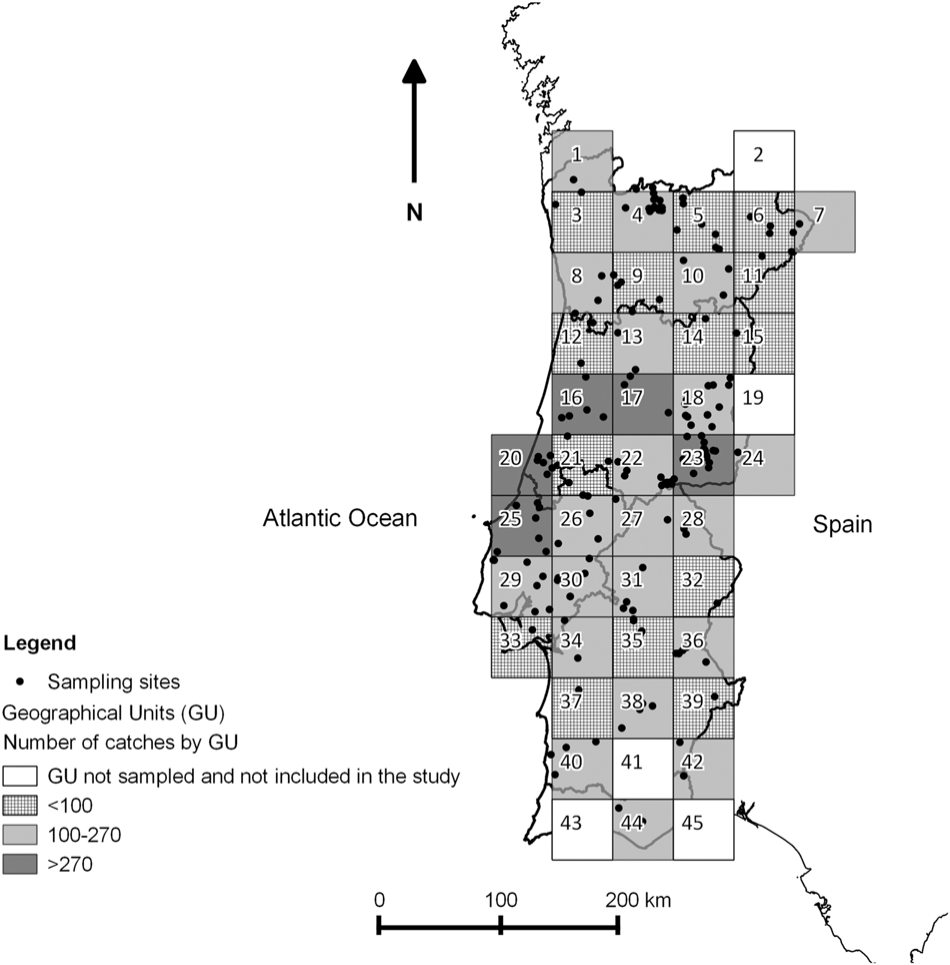
Design of the Entomological Surveillance Programme through mainland Portugal, showing sampling effort by GU.

The ESP was designed to conform to the requirements of European Council directive 2000/75/EC, and it is similar to that used for previous *Culicoides* surveillance programmes in Italy [20] and Greece [21] Mainland Portugal was divided into 45 geographical units (GUs) each 50km by 50km (Fig 2). Within each GU, suitable farms were identified using the national database of farms and farmers were contacted by the National Authority for Animal Health (Direcção-Geral de Veterinária, DGV; http://www.dgv.min-agricultura.pt/) and asked if they agreed to participate in the national programme for bluetongue control. All the farmers gave permission to place the traps in their farms, as it was explained that it would be a good contribution for the protection of animal health. To be eligible for inclusion, farms had to be located at a minimum distance of 10km from other sampled holdings and at least 2.5km from the coast, and contain a minimum of five horses or ruminants (preferably cattle). Recruited farms were not permitted to apply insecticides for the duration of the programme. Four GUs in mainland Portugal were excluded from the study due to low livestock densities (GUs 2, 41, 43 and 45), and the number of farms recruited in each of the remaining GUs varied between 1 and 18. Additional farms were recruited close to the edges of the historically affected zone to allow more precise monitoring of these regions.

On each of the farms in the study, Center for Disease Control (CDC) miniature light traps (model 1212; John W.Hock, Gainesville, FL, US) fitted with a 4W UV light and a suction fan were placed outdoors at a height of 1.7–2.0 m and at a distance of up to 30m from the livestock. Insects were collected into plastic shatterproof beakers containing approximately 375ml of 70% ethanol and 125ml of ethylene glycol, giving a final volume of 500 ml. The traps were powered using a 6V battery and were turned on by an LCS-2 photoelectric cell that activated the trap at dusk and deactivated it at dawn. Samples were collected and traps reset by technical staff of the Regional Veterinary Services. Although the ESP formally started in May 2005, six months after the 2004 outbreak, collections did not begin until September 2005 as a consequence of the time required to train staff and to purchase and distribute suitable equipment and consumables. A higher number of catches were performed during spring and autumn to better define the vector free period, which is defined by the total absence of *C*. *imicola* specimens and less than five parous/pigmented *Culicoides* per trap. This is important for the establishment of vector free areas and for the implementation of movement restrictions [22].

Samples were sent to the Interdisciplinary Centre of Research in Animal Health (CIISA) at the University of Lisbon for identification. Upon arrival they were transferred to 96% ethanol and identified using morphological keys based on wing patterns [23–25]. This permitted the identification of suspected vector species including *C*. *imicola*, members of the group Pulicaris (*C*. *pulicaris* (L.); *C*. *lupicaris*, *C*. *newsteadi* Austen, C. *punctatus* (Meigen)) and members of the Obsoletus group (*C*. *obsoletus*sensu stricto (Meigen); *C*. *chiopterus* (Meigen); *C*. *dewulf* Goetghebuer;, *C*. *scoticus* Downes and Kettle; *C*. *montanus* Shakirzjanova), as well as *C*. *circumscriptus* Kieffer, *C*. *maritimus* Kieffer and *C*. *univittatus* Vimmer [26,27]. Other *Culicoides* were stored for more detailed taxonomic studies. Sex and the physiological condition of females (nulliparous/unpigmented, blood-fed, gravid, or parous/pigmented) were also recorded. In the case of large samples, a sub-sample of 2.5% (for samples containing more than 5,000 individuals) or 25% (for samples containing fewer than 5,000 individuals but more than 2,000) was analysed, following published guidelines [28]. Results were provided to the DGV in real time to inform policy makers in relation to the BTV situation, specifically animal movement restrictions and the prioritization of vaccination areas.

Trapping sites were geo-referenced with a Trip Tracker GPS system (Amaryllo, Netherlands) wherever possible; when geographical coordinates could not be obtained, coordinates of the centroid of the parish were used.

At each insect trapping site, environmental factors which might be expected to influence the presence/absence of BTV vectors were also noted.

Associated climate data, monthly diurnal temperature range (DTR), frost days (FRS), precipitation (PRE), and median temperature (TMP), were extracted from the relevant pixels of the Climate Research Unit high-resolution gridded climatology (CRU TS3.10) raster layer (29) to form the basis of the analyses below. The primary variables (DTR, PRE and TMP) are monthly based values, derived from archives of climate station records from January 1901 to December 2009. Secondary variable (FRS) is entirely derived from the primary variables [29].

Variables were zero-centred and scaled to yield a standard deviation of one. However, minimum temperature (TMN), maximum temperature (TMX), potential evapotranspiration (PET), vapour pressure deficit (VAP) and rainday counts (WET) were excluded due to high levels of correlation.

### Statistical analysis

**Fig 3:**
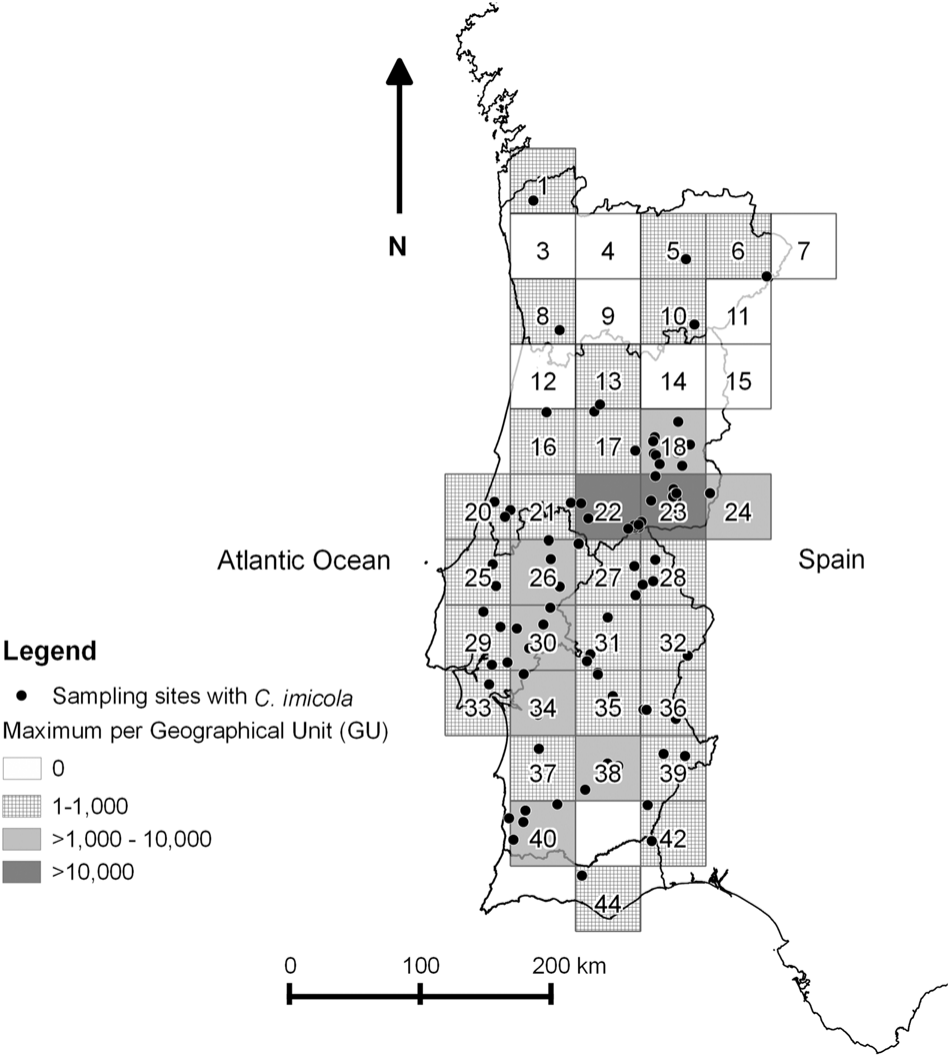
Sampling sites with presence of *C*. *imicola* Kieffer and maximum level collected per GU, during ESP.

**Fig 4:**
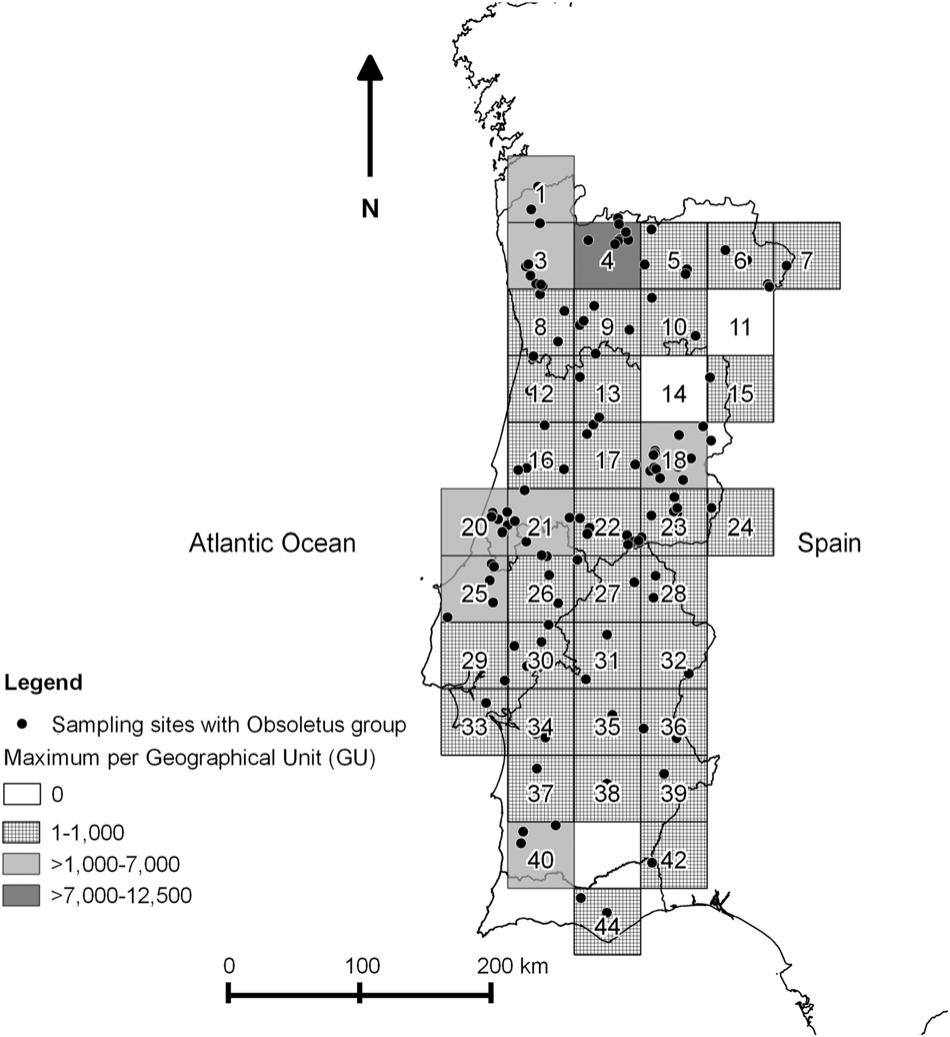
Sampling sites with presence of the Obsoletus group (*C*. *obsoletus* (Meigen); *C*. *chiopterus* (Meigen); *C*. *dewulfi* Goetghebuer; *C*. *scoticus* Downes and Kettle; *C*. *montanus* Shakirzjanova) and maximum level collected per GU, during ESP.

**Fig 5:**
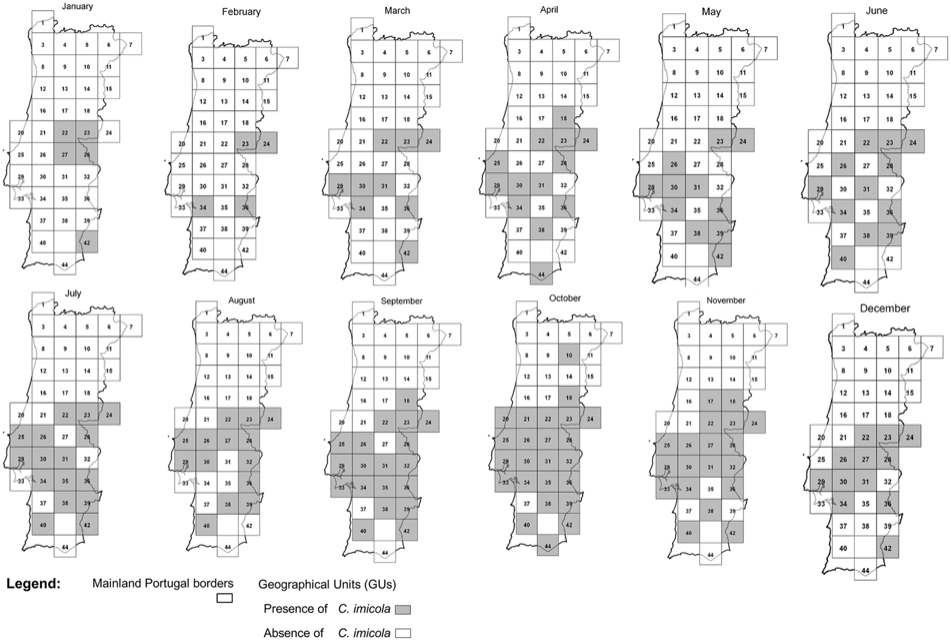
Presence and absence of C. *imicola* Kieffer per GU by month.

**Fig 6:**
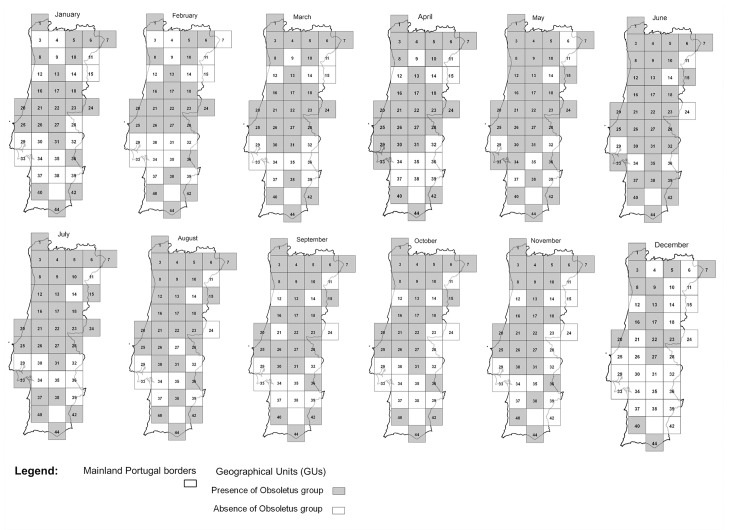
Presence and absence of the Obsoletus group (*C*. *obsoletus* (Meigen); *C*. *chiopterus* (Meigen); *C*. *dewulfi* Goetghebuer; *C*. *scoticus* Downes and Kettle; *C*. *montanus* Shakirzjanova) per GU by month.

The datasets used in this study included all catches made within the Portuguese national entomological surveillance programme between 2005 and 2010 (S1 Table). All maps were designed using Quantum GIS version 1.8.0 [30]. A map (Fig 2) identifies all sampling sites and the number of catches performed in each GU for all the study period, representing the sampling effort by GU. Two other figures represent the sampling sites where *C*. *imicola* (Fig 3) or Obsoletus group (Fig 4) were captured, as well as the overall maximum capture of each species by GU in the five years in study. Also, presence or absence for each species in each GU was plotted by month (Fig 5 and Fig 6). Shapefiles for the Portuguese administrative areas from the “Carta Administrativa Oficial de Portugal” (CAOP) were downloaded from the Portuguese Geographical Information (Informação Geográfica; iGeo) repository (http://www.igeo.pt/produtos/cadastro/caop/caop_vigor.htm).

Wilcoxon two-sample test was used to compare pairwise the northern, center and southern groups of GUs as regard the maximum captures of *C*. *imicola* and Obsoletus group, during all the five years in study. A p-value less than 5% was chosen as significant level.

To determine the relative importance of season and climatological factors in the likelihood of collecting specimens from the Obsoletus group or *C*. *imicola*, a generalized linear mixed model (GLMM) approach was used with binomial errors and a logit link function in which collection site was represented as a random effect.

A generalised model was used because we are modelling a binomial response (presence or absence of the vector species or group) and a mixed model was necessary because repeated measures were taken at the same site, introducing random effects. GLMMs combine the properties of linear mixed models, which incorporate random effects, and generalised linear models, which handle nonnormal response data by using link functions [31].

The GLMM was implemented in R version 3.1.0 [32] using the function “bglmer” in the ‘blme’ package. The full models included the factors ‘month’, ‘DTR’, ‘FRS’, ‘PRE’ and ‘TMP’, with ‘SITE’ as a random effect. Quadratic terms for ‘PRE’ and ‘TMP’ were also included because the biological effects of these variables might reasonably be expected to exhibit local maxima or minima within the observed range and for the species studied. Backward stepwise elimination of variables was then implemented by dropping each term individually and performing a likelihood ratio test on the difference in the Akaike Information Criterion (AIC) using the function ‘dropterm’ from the ‘MASS’ package.

## Results

### Entomological surveillance programme

Two-hundred and seventy-four farms were sampled between September 2005 and December 2010 as part of the ESP, of which 212 (77.4%) were in mainland Portugal. In addition to the four GUs already mentioned where no farms were recruited due to low livestock densities, no collections were received from the farm in GU 19. The five GUs with the largest number of catches were GUs 20, 23, 17, 25 and 16, which accounted for 26% of the total number of catches (Fig 2).

A total of 5,650 catches were performed in mainland Portugal. The highest number of catches was registered in the central region (57.7% of the total, 3,261/5, 650 catches) and the lowest number of catches occurred in the northern region of the country (21.6%, 1,221/5,650 catches).

The number of collections by month varied from a minimum of 37 catches (0.65% of total) in December 2010 to 135 catches (2.4% of total) in April 2008. The months with the highest number of catches were April 2008, March 2009, March 2007 and July 2008. Regarding the season of the year, the highest number of catches occurred during autumn (1,587 catches, 28% of total) while the lowest number of catches was verified in winter (1,186 catches, 21% of total).

*Culicoides* were present in 62.6% of valid catches (3,632/5,800) and 28 different species were identified. The potential vector species most commonly caught were *Culicoides imicola* and members of the Obsoletus group [33]. *C*. *imicola* was present in 74.8% of the collections, while specimens from Obsoletus group were present in only 7.7%. Group Pulicaris was present in only 0.1% of the collections.

The central region of the country accounted for the highest catches of *C*. *imicola*. The highest maximum values of specimens captured, 107,880 and 450,800 specimens were reported respectively, in GUs 22 and 23 (Fig 3). GU23 was also the highest altitude location where *C*. *imicola* was collected, 1,654m above sea level. *C*. *imicola* was collected less frequently and in smaller quantities in the north of the country, where the maximum catch was three individuals (in GU 10). The northernmost collection containing *C*. *imicola* was made at 41.92°N.

Catches of Obsoletus group were highest in the north and centre of Portugal. GUs 4, 20 and 21 had the highest catches, with maximum catches of 12,120 specimens in GU4, of 6,179 specimens in GU21 and 6,160 specimens in GU20. The smallest catches of this group were observed in the south of the country, particularly in GUs 29 and 32 where collections only reached a maximum of one specimen per catch (Fig 4).

*C*. *imicola* was collected in fewer GUs than the Obsoletus groups. Comparing our data with the most recent previous survey of *Culicoides* in Portugal dating from 2001–2002 [6], we detected *C*. *imicola* in five more GUs, mainly in the northern regions (GU number 1, 5, 8, 16 and 17), although no specimens were found in GU11 where the species had previously been reported.

The maximum number of *C*. *imicola* captures per GU was lower in the northern region when compared with both centre and southern regions (*Wilcoxon* two-sample test, p < 0.001). There were no differences between *C*. *imicola* collections in the centre and south regions. For Obsoletus group, the same test did not shown differences between the three pairs of regions (*Wilcoxon* two-sample test, p >0.05).

**Fig 7:**
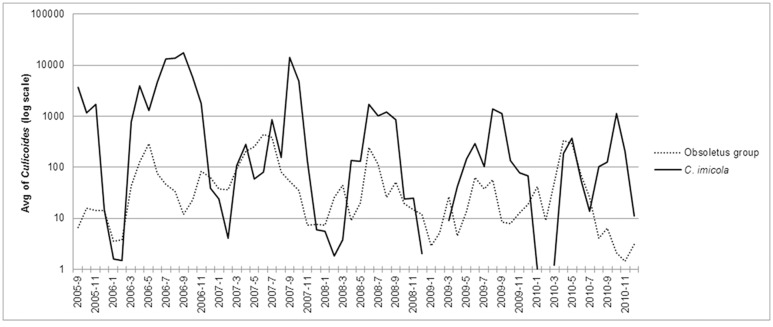
Seasonality of *C*. *imicola* and Obsoletus group between September 2005 and December 2010.

Fig 7 shows the monthly average of *C*. *imicola* and Obsoletus group collections in the mainland territory between September 2005 and December 2010.

In general, *C*. *imicola* activity began between March and April, but between 2007 and 2008 there were collections of a limited number of *C*.*imicola* in January. The largest collections of this species were made in July, August and September 2006 and September and October 2007, with collections exceeding 100,000 specimens.

In the centre of the country peak activity was observed between September and October, while in the southern regions the period of highest activity of *C*. *imicola* extended between September and November except in 2010 when, as a result of the lower number of catches performed, it reached the highest levels in April.

In the southern region, the highest abundances of *C*. *imicola* occurred in GUs 34, 38 and 40.

Although in small numbers, seasonal activity of Obsoletus group was detected as early as January. In the northern regions this species reached the highest abundances between May and August (commonly in GUs 1, 3 or 4), while in the central regions this occurred between May and June (in GUs 18, 20 or 21). In the southern regions, Obsoletus group typically reached peak abundance between April to May (in GUs 40, 42 or 44). The largest catches of this group occurred in May 2005, June 2007, June 2008 and April 2010, with collections exceeding 5,000 specimens. The largest catch in the study period was in June 2008, when a single catch reached a maximum of 12,120 specimens.

The abundances of *C*. *imicola* and the Obsoletus group were lowest during winter (December-January-February). This was more pronounced for *C*. *imicola*, however, in both cases a proportion of the females collected during winter were parous, ranging from 20.3% (in 2006) to 76.4% (in 2009) for *C*. *imicola* and from 13.3% (2005) to 35.3% (2007) for the Obsoletus group.

The results of the generalized linear mixed model are shown in Table 1 (*C*. *imicola* activity) and Table 2 (the same analysis for the Obsoletus group). For *C*. *imicola*, the most important variables were month (Fig 5 —presence and absence of *C*. *imicola* by month), diurnal temperature range (DTR), the number of frost days (FRS) and median monthly temperature (TMP). For the Obsoletus group, the most important factors were month (Fig 6 —presence and absence of the Obsoletus group by month), diurnal temperature range, and linear and quadratic terms for median monthly temperature (TMP).

**Table 1:** Results of a generalized linear mixed model for the presence of *Culicoides imicola* Kieffer in light trap catches.

Parameter	Value	Standard error	p-value
**Intercept**	-7.97995	0.83901	<2*10^-16^
**Month**	-	-	<2*10^-16^ †
** Feb**	-0.44552	0.56304	0.42878
** Mar**	0.16140	0.47564	0.73437
** Apr**	0.14409	0.51610	0.78009
** May**	-1.15147	0.60783	0.05817
** Jun**	-1.82478	0.75305	0.01538
** Jul**	-0.90596	0.80653	0.26132
** Aug**	-0.20352	0.81833	0.80359
** Sep**	0.71795	0.71765	0.31711
** Oct**	1.80707	0.57538	0.00169
** Nov**	2.35728	0.45139	1.77*10^-7^
** Dec**	1.76687	0.44626	7.52*10^-5^
**DTR**	-0.12758	0.05339	0.01687
**FRS**	0.14208	0.05739	0.01330
**TMP**	0.30734	0.05892	1.83*10^-7^

^†^ indicates p-value calculated from the likelihood ratio of the AICs of the models with and without the categorical variable; other p-values as obtained from bglmer.

**Table 2:** Results of a generalized linear mixed model for the presence of Obsoletus group (*C*. *obsoletus* (Meigen); *C*. *chiopterus* (Meigen); *C*. *dewulfi* Goetghebuer; *C*. *scoticus* Downes and Kettle; *C*. *montanus* Shakirzjanova) in light trap catches.

Parameter	Value	Standard error	p-value
**Intercept**	-3.917327	0.564970	4.1*10^-12^
**Month**	-	-	2.2*10^-16^ †
** Feb**	0.230018	0.211661	0.2772
** Mar**	0.521081	0.217637	0.0167
** Apr**	0.591416	0.254017	0.0199
** May**	1.334120	0.304999	1.22*10^-5^
** Jun**	1.870122	0.396932	2.46*10^-6^
** Jul**	1.772989	0.440798	5.77*10^-5^
** Aug**	1.162644	0.453682	0.0104
** Sep**	0.263910	0.389301	0.4978
** Oct**	-0.340541	0.307223	0.2677
** Nov**	-0.312666	0.227004	0.1684
** Dec**	-0.095133	0.232401	0.6823
**DTR**	-0.067006	0.030730	0.0292
**TMP**	0.438969	0.072722	1.58*10^-9^
**TMP (quadratic term)**	-0.013189	0.002218	2.74*10^-9^

^†^ indicates p-value calculated from the likelihood ratio of the AICs of the models with and without the categorical variable; other p-values as obtained from bglmer.

## Discussion

### Descriptive analysis of catches and *Culicoides* spp. captures in Portugal

The data presented here represents the results of the largest survey of *Culicoides* in mainland Portugal to date. The primary purpose of the surveillance was to define the range of potential vectors of BTV, AHSV, and other Culicoides-borne viruses in order to define appropriate areas for movement restriction during outbreaks, to define seasonal activity periods by region in order to implement appropriate and proportionate seasonal movement restrictions and animal vaccination. Mass vaccination programmes should take into account the presence of active potential *Culicoides* vectors. Effective animal immunization, should preferentially take place before vector seasonal activity starts, being also important to minimize the risks of infection of the insects with a vaccinal BTV virus strain, if vaccination with live vaccine is used, as occurred initially after BTV 4 emergence in PT, in 2005 [13].

Secondary objectives were to collect time-series data of presence and abundance of each major vector group in order to monitor and analyse potential changes in their distribution and to improve control measures.

Based on our data, *C*. *imicola* is the most prevalent *Culicoides* species in Portugal, followed by members of the Obsoletus group. This is in agreement with previous studies [6]. Specimens from the group Pulicaris, pottencially important BTV vectors in Europe, were found in very low numbers. For this reason *C*. *imicola*, followed by species of the Obsoletus group, are considered as the most likely potential vectors of BTV in Portugal [2,6].

*C*. *imicola* is more abundant in the central and southern regions of Portugal, but was virtually absent from the north of the country, while the Obsoletus group is distributed more homogenously through the country. These differences are likely to represent previously recognized differences on the climatic and habitat preferences for each species/group [34].

The geography of continental Portugal is influenced both by Atlantic and Mediterranean, with the first dominating the northern region and the second dominating the south of the country.

Tagus divides the mountainous regions of the north, characterized by forests crossed by deep valleys, from the southern regions, with lower elevations and vast lowlands cultivated with Mediterranean species as cork oaks, fig and olive trees and vines.

The climate is temperate oceanic, with hot summers and cold winters. The northwest has a maritime climate, with short, cool summers and mild winters. In the northeast the climate is more continental, with sharper contrasts between the seasons. The central part of the country has hot summers and mild, rainy winters, and the south has a dry climate with long, hot summers. Lisbon, Alentejo and Algarve have long and hot summers with temperatures range between 35°C and 40°C. In the northern regions, the average air temperature (annual average values) range from less than 7.5 to 16°C in the inner regions. In the center and southern regions the values range between 12.5°C to 16°C and more than 17.5°C in the south. Average annual rainfall ranges from over 1000mm in the northern regions and 700–500 and even less than 400mm in the center and southern regions [35].

The higher abundance of *C*. *imicola* in the southern region is probably due to its preference for breeding in areas where soil is moist and nutrient-rich and with full exposure to sunlight, characteristics which are more commonly found in the centre and south of Portugal. Conversely Obsoletus group is less dependent on soil type [34].

The geographic ranges of most insect species are influenced by temperature and the low temperatures tend to be more significant than high temperatures as determinants of distribution [36]. There is some evidence that the northern limit of *C*. *imicola* in Europe is determined by temperature [36]. Possibly the lower minimum temperatures in the northern regions, which are in average 2.9°C lower than minimum temperatures in southern Portugal [37], can explain the absence of *C*. *imicola*.

The present study reports the most northerly collection of *C*. *imicola* in Portugal ever recorded (41.92°N). In the Iberian Peninsula as a whole, the most northerly collection of *C*. *imicola* remains 42.3°N [38]. Also reported in the study described here was the highest altitude collection of *C*. *imicola* in the Iberian Peninsula, at 1,654m. This compares to previous records of 1,261m in Portugal [6] and 1,100m in Spain [39]. However, the extremely low numbers caught suggest that these specific habitats may be of borderline suitability for the species, and the specimens caught could potentially have been transported from more suitable regions by wind [40].

The abundances of *C*. *imicola* and Obsoletus group were lowest during winter (December-January-February). This was more pronounced for *C*. *imicola*. However, in both cases a proportion of the females collected during winter were parous. In some parts of northern Europe it has also been demonstrated that active adult *Culicoides*, including species of the Obsoletus group, can be collected in small numbers in every month throughout the winter period (i.e. November-March) [34]. The continuous activity of adult *Culicoides* throughout the year, as demonstrated to occur in central Portugal during this study, could facilitate the persistence of BTV as long as temperatures are adequate for virus replication or if adult survival is prolonged during cold periods, permitting the survival of infected vectors [41].

Regarding winter activity of potential vector species, one particular region in the centre of the country (Idanha a Nova, within GU23-24), deserves attention. This area, which registered continuously large catches of *C*. *imicola*, also demonstrated year-round *C*. *imicola* activity. Further studies of this region are planned.

The collections of *C*. *imicola* started to increase in the middle of spring (between March to April), peaked between September and November, decreased at the beginning of winter and remained at low levels or were null during this period. In contrast, the Obsoletus group did not show such a marked seasonality. Numbers of Obsoletus group specimens collected started to increase in the beginning of spring, peaked earlier, predominantly between May and August, and showed the lowest values during the winter but some activity was observed throughout the year. In the northern regions species of the Obsoletus group remained active until the end of summer or beginning of the autumn season.

Statistical analysis of these data suggested that for both *C*. *imicola* and the Obsoletus group, the model fit was significantly worsened by failing to include month as a descriptor of activity. In both cases higher median monthly temperatures and lower diurnal temperature ranges significantly increased the probability of detectable *Culicoides* activity. The model for *C*. *imicola* also indicated a small but significant positive effect of the number of frost days, and the model for the Obsoletus group indicated that the effect of median temperature was best modelled as a quadratic rather than a linear effect. Since these indicate climatic effects beyond those accounted for by the time of year, the positive effect of median temperature combined with the negative effect of diurnal temperature range would suggest that vector activity of *Culicoides* is more likely during months which are consistently warmer than normal for the time of year. However, the positive influence of frost days on *C*. *imicola* activity appears to contradict this statement and further investigation of this apparent relationship is needed.

Previous studies have suggested that the activity of Obsoletus group, the probability for the observation of at least one biting midge in the light trap is best explained by season and daytime land surface temperature (dLST), which are likely to govern the commencement of the *Culicoides* adult active season [42]. Regarding *Culicoides* abundance the variables determining higher numbers of *Culicoides* per trap catch were related to temperature (dLST), whereas wind and the evapotranspiration index were associated with lower *Culicoides* counts [42].

Factors most likely to affect light trap collection success that were not measured in this study include cloud cover, wind speed and humidity. These variables are often omitted from studies of this type due to the additional expense of measuring them, the difficulty of predicting these variables, and the high levels of temporal (in the case of wind) and spatio-temporal (in the case of humidity) variability. However, we strongly encourage future researchers to consider measuring these to facilitate the development of accurate models of *Culicoides* activity.

Most previous studies of *Culicoides* distribution and activity in southern Europe have considered a combination of several types of factors, the majority including spatial, topographic, soil, climatic and host factors,however, their results are not always consistent. This may be due to the different combination of factors analyzed in each study and the various statistical approaches used.

Acevedo *et al*., 2010 used a combination of spatial, topographic, soil, climatic and host predictors to determine the spatial pattern of abundance of *C*. *imicola* in peninsular Spain, using variation partitioning techniques [43]. They found that host and topoclimatic factors explained a high percentage (80%) of the variation, and within topoclimatic factors, precipitation and its seasonality and, to a lesser extent, temperature, were the most important factors in explaining *C*. *imicola* distribution.

In Wittman *et al*., 2001, temperature-related variables were the variables with the highest explanatory power for *C*. *imicola* distribution models [44]. Precipitation was also important, which may agree with the requirement of *C*. *imicola* for humid organically enriched soil as breeding sites [45]. Remotely sensed variables of land surface temperature and the normalized difference vegetation index (NDVI), correlates of temperature and soil moisture, respectively, have been shown to be valuable predictors of regional and continental scale patterns in BT and its *Culicoides* vectors [19,46–48].

Purse *et al*., 2012 investigated the influence of landscape, host and remotely-sensed climate factors on local abundance of Obsoletus group and group Pulicaris in Scotland, within a hierarchical generalized linear model framework [49]. In this study, local-scale abundance patterns were best explained by models combining host, landscape and climate factors. The most important single set of predictors was climate (land surface temperatures). In the specific case of the Obsoletus group, neither models of single or combined set of predictors could effectively explain overall abundance patterns [49].

A study of *C*. *imicola* and of Obsoletus group in Italy using discriminant analysis [50] identified that both biotic and abiotic (topography, temperature and aridity index) factors were important in explaining the total variability of *C*. *imicola* and species from Obsoletus group. However, the Obsoletus group showed a higher tolerance and a capacity to survive to more variable conditions than *C*. *imicola*, which is similar to the conclusions we have drawn from our current model.

Although there are some discrepancies between the findings of these studies, most agree that variables related to availability of moisture (precipitation, evapotranspiration and NDVI), and temperature-related variables are the best predictors for *Culicoides* activity. Further investigation and direct comparison of published datasets and models is difficult because they are not typically shared in a way which allows other researchers free access to attempt to replicate results or apply alternative statistical approaches. We hope that by sharing our data and methods in a transparent fashion we will help to facilitate future meta-analyses of the relationship between environment and *Culicoides* activity.

## Conclusion

The Portuguese national entomological surveillance programme (ESP) made 5,650 valid light-trap catches on 212 farms in mainland Portugal between 2005 and 2010. This period also covered substantial BTV activity in mainland Portugal. The data presented here extend the known range of *C*. *imicola* in Portugal, improve our understanding of the seasonal activity of *Culicoides* vectors of livestock disease and potential climatic factors in *Culicoides* activity, and improve our knowledge of the activity of important vector species during the winter period. We also provide the data collected (S1 Table) to support future research by other groups. *Culicoides* are important vectors for a range of pathogens causing diseases with veterinary and public health importance including BTV, AHSV, epizootic haemorrhagic disease (EHDV), filarial diseases and the recently-discovered SBV. We therefore hope that our data and analyses can be useful for future studies of *Culicoides* and *Culicoides*-borne disease and are able to support the design of strategies to prevent their spread in the country.

## Supporting Information

S1 Table Datasets of all valid catches made within the Portuguese national entomological surveillance programme between 2005 and 2010. (CSV)

## References

[1] Maan S, Maan NS, Nomikou K, Veronesi E, Bachanek-Bankowska K, Belaganahalli MN, et al Complete Genome Characterisation of a Novel 26th Bluetongue Virus Serotype from Kuwait. Beer M, editor. PLoS ONE. 2011 10 21;6(10):e26147 10.1371/journal.pone.0026147 22031822 PMC3198726

[2] Mellor PS, Boorman J, Baylis M. Culicoides biting midges: their role as arbovirus vectors. Annu Rev Entomol. 2000;45(1):307–40. 10761580 10.1146/annurev.ento.45.1.307

[3] Backx A, Heutink R, van Rooij E, van Rijn P. Transplacental and oral transmission of wild-type bluetongue virus serotype 8 in cattle after experimental infection. Vet Microbiol. 2009 9;138(3–4):235–43. 10.1016/j.vetmic.2009.04.025 19419822

[4] Dal Pozzo F, De Clercq K, Guyot H, Vandemeulebroucke E, Sarradin P, Vandenbussche F, et al Experimental reproduction of bluetongue virus serotype 8 clinical disease in calves. Vet Microbiol. 2009 5;136(3–4):352–8. 19128895 10.1016/j.vetmic.2008.11.012

[5] Mellor PS, Carpenter S, Harrup L, Baylis M, Mertens PPC. Bluetongue in Europe and the Mediterranean Basin: History of occurrence prior to 2006. Prev Vet Med. 2008 10;87(1–2):4–20. 10.1016/j.prevetmed.2008.05.007 18619694

[6] Capela R, Purse BV, Pena I, Wittman EJ, Margarita Y, Capela M, et al Spatial distribution of Culicoides species in Portugal in relation to the transmission of African horse sickness and bluetongue viruses. Med Vet Entomol. 2003;17(2):165–77. 12823834 10.1046/j.1365-2915.2003.00419.x

[7] Carpenter S, Wilson A, Mellor PS. Culicoides and the emergence of bluetongue virus in northern Europe. Trends Microbiol. 2009 4;17(4):172–8. 10.1016/j.tim.2009.01.001 19299131

[8] Darpel KE, Batten CA, Veronesi E, Williamson S, Anderson P, Dennison M, et al Transplacental Transmission of Bluetongue Virus 8 in Cattle, UK. Emerg Infect Dis. 2009 12;15(12):2025–8. 10.3201/eid1512.090788 19961692 PMC3044536

[9] EFSA Panel on Animal Health and Welfare (AHAW). Scientific Opinion of the AHAW Panel: Bluetongue serotype-8. EFSA Journal. 2011;51 pp.

[10] Menzies FD, McCullough SJ, McKeown IM, Forster JL, Jess S, Batten C, et al Evidence for transplacental and contact transmission of bluetongue virus in cattle. Vet Rec. 2008;163(7):203–9. 18708653 10.1136/vr.163.7.203

[11] Wilson A, Darpel K, Mellor PS. Where does bluetongue virus sleep in the winter? PLoS Biol. 2008;6(8):e210 10.1371/journal.pbio.0060210 18752350 PMC2525685

[12] Sellers RF, Taylor WP. Epidemiology of bluetongue and the import and export of livestock, semen and embryos. Bull Off Int Epizoot. 1980;92:587–92.

[13] Barros SC, Ramos F, Luís TM, Vaz A, Duarte M, Henriques M, et al Molecular epidemiology of bluetongue virus in Portugal during 2004–2006 outbreak. Vet Microbiol. 2007 9 20;124(1–2):25–34. 17521832 10.1016/j.vetmic.2007.04.014

[14] Portas M, Boinas FS, Sousa JOE, Rawlings P. African horse sickness in Portugal: a successful eradication programme. Epidemiol Infect. 1999 10;123(2):337–46. 10579455 10.1017/s0950268899002897 PMC2810767

[15] Direcção Geral de Alimentação e Veterinária (DGAV). Programa de erradicação, controlo e vigilância da Língua Azul. 2012.

[16] European Commission (EC). Animal Disease Notification System. Report summary [Internet]. 2012. Available from: http://ec.europa.eu/food/animal/diseases/adns/adns_outbreaks_per_disease_en.pdf.

[17] Direcção Geral de Alimentação e Veterinária (DGAV). Bluetongue. Situation in Portugal. 2014.

[18] Hoffmann B, Scheuch M, Höper D, Jungblut R, Holsteg M, Schirrmeier H, et al Novel Orthobunyavirus in Cattle, Europe, 2011. Emerg Infect Dis. 2012 3;18(3):469–72. 10.3201/eid1803.111905 22376991 PMC3309600

[19] Tatem AJ, Baylis M, Mellor PS, Purse BV, Capela R, Pena I, et al Prediction of bluetongue vector distribution in Europe and north Africa using satellite imagery. Vet Microbiol. 2003 12 2;97(1–2):13–29. 14637035 10.1016/j.vetmic.2003.08.009

[20] Goffredo M, Meiswinkel R. Entomological surveillance of bluetongue in Italy: methods of capture, catch analysis and identification of Culicoides biting midges. Vet Ital. 2004 Sep;40(3):260–5. 20419674

[21] Patakakis MJ, Papazahariadou M, Wilson A, Mellor PS, Frydas S, Papadopoulos O. Distribution of Culicoides in Greece. J Vector Ecol. 2009 11 12;34(2):243–51. 10.1111/j.1948-7134.2009.00033.x 20836829

[22] European Commission (EC). Commission Regulation (EC) No 1266/2007 of 26 October 2007 on implementing rules for Council Directive 2000/75/EC as regards the control, monitoring, surveillance and restrictions on movements of certain animals of susceptible species in relation to bluetongue. Commission Regulation (EC) 1266/2007 Oct 26, 2007.

[23] Rawlings P. A key, based on wing patterns of biting midges (genus Culicoides Latreille-Diptera: Ceratopogonidae) in the Iberian Peninsula, for use in epidemiological studies. Graellsia. 1996;52(11):57–71.

[24] Delécolle JC. Nouvelle contribuition à l’étude systématique et iconographique dés espèces du genre Culicoides (Diptera: Ceratopogonidae) du Nord-Est de la France. Université Louis Pasteur; 1985 10.1016/j.crvi.2014.04.006

[25] Pena I. Contribuição para o estudo da sistemática, biologia e ecologia dos Culicoides (Diptera, Ceratopogonidae) de Portugal Sua importância médica e veterinária. Universidade da Madeira; 2003.

[26] Garros C, Balenghien T, Carpenter S, Delécolle J-C, Meiswinkel R, Pédarrieu A, et al Towards the PCR-based identification of Palaearctic Culicoides biting midges (Diptera: Ceratopogonidae): results from an international ring trial targeting four species of the subgenus Avaritia. Parasit Vectors. 2014;7(1):223. 24884950 10.1186/1756-3305-7-223 PMC4024274

[27] Ayllón T, Nijhof AM, Weiher W, Bauer B, Allène X, Clausen P-H. Feeding behaviour of Culicoides spp. (Diptera: Ceratopogonidae) on cattle and sheep in northeast Germany. Parasit Vectors. 2014;7(1):34. 24438698 10.1186/1756-3305-7-34 PMC3896851

[28] Van Ark H, Meiswinkel R. Subsampling of large light trap catches of Culicoides (Diptera: Ceratopogonidae). Onderstepoort J Vet Res. 1992 9;59(3):183–9. 1437020

[29] Harris I, Jones PD, Osborn TJ, Lister DH. Updated high-resolution grids of monthly climatic observations—the CRU TS3.10 Dataset: UPDATED HIGH-RESOLUTION GRIDS OF MONTHLY CLIMATIC OBSERVATIONS. Int J Climatol. 2014 3 15;34(3):623–42.

[30] QGis [Internet]. Available from: http://www.qgis.org/

[31] Bolker BM, Brooks ME, Clark CJ, Geange SW, Poulsen JR, Stevens MHH, et al Generalized linear mixed models: a practical guide for ecology and evolution. Trends Ecol Evol. 2009 3;24(3):127–35. 10.1016/j.tree.2008.10.008 19185386

[32] The R Project for Statistical Computing [Internet]. Available from: http://www.r-project.org/

[33] Ramilo DW, Diaz S, Pereira da Fonseca I, Delécolle J-C, Wilson A, Meireles J, et al First Report of 13 Species of Culicoides (Diptera: Ceratopogonidae) in Mainland Portugal and Azores by Morphological and Molecular Characterization. PLoS ONE. 2012 Abril;7(4):e34896 10.1371/journal.pone.0034896 22536340 PMC3334969

[34] EFSA Panel on Animal Health and Welfar e. Scientific Opinion of the Panel on Animal Health and Welfar e on a request from the European Commission (DG SANCO) on Bluetongue. The EFSA Journal. 2008;1–70.

[35] Ferreira AMPJ. Dados Geoquímicos de Base de Sedimentos Fluviais de Amostragem de Baixa Densidade de Portugal Continental: Estudo de Factores de Variação Regional [Internet] [Tese de doutoramento]. [Aveiro]: Universidade de Aveiro; 2000. Available from: http://hdl.handle.net/10400.9/542

[36] Wittmann EJ, Baylis M. Climate Change: Effects on Culicoides-Transmitted Viruses and Implications for the UK. Vet J. 2000 9;160(2):107–17. 10985802 10.1053/tvjl.2000.0470

[37] FFMS. Pordata—Temperatura média do ar (média anual) em Portugal [Internet]. Pordata. 2014. Available from: http://www.pordata.pt/Portugal/Temperatura+media+do+ar+(media+anual)-1067

[38] Sarto i Monteys V, Ventura D, Pagès N, Aranda C, Escosa R. Expansion of Culicoides imicola, the main bluetongue virus vector in Europe, into Catalonia, Spain. Vet Rec. 2005 3 26;156(13):415–7. 15816196 10.1136/vr.156.13.415

[39] Calvete C, Miranda MA, Estrada R, Borras D, Monteys VSI, Collantes F, et al Spatial distribution of Culicoides imicola, the main vector of bluetongue virus, in Spain. Vet Rec. 2006;158(4):130–1. 16443839 10.1136/vr.158.4.130

[40] García-Lastra R, Leginagoikoa I, Plazaola JM, Ocabo B, Aduriz G, Nunes T, et al Bluetongue Virus Serotype 1 Outbreak in the Basque Country (Northern Spain) 2007–2008. Data Support a Primary Vector Windborne Transport. Gubbins S, editor. PLoS ONE. 2012 3 30;7(3):e34421 10.1371/journal.pone.0034421 22479628 PMC3316701

[41] Takamatsu H, Mellor P., Mertens PP., Kirkham PA, Burroughs JN, Parkhouse RME. A possible overwintering mechanism for bluetongue virus in the absence of the insect vector. J Gen Virol. 2003 1 1;84(1):227–35. 12533719 10.1099/vir.0.18705-0

[42] Scolamacchia F, Van Den Broek J, Meiswinkel R, Heesterbeek JAP, Elbers ARW. Principal climatic and edaphic determinants of *Culicoides* biting midge abundance during the 2007–2008 bluetongue epidemic in the Netherlands, based on OVI light trap data: *Local abundance of Dutch* Culicoides. Med Vet Entomol. 2014 6;28(2):143–56. 10.1111/mve.12028 24148154

[43] Acevedo P, Ruiz-Fons F, Estrada R, Márquez AL, Miranda MA, Gortázar C, et al A Broad Assessment of Factors Determining Culicoides imicola Abundance: Modelling the Present and Forecasting Its Future in Climate Change Scenarios. Cornell SJ, editor. PLoS ONE. 2010 12 6;5(12):e14236 10.1371/journal.pone.0014236 21151914 PMC2997795

[44] Wittmann EJ, Mellor PS, Baylis M. Using climate data to map the potential distribution of Culicoides imicola (Diptera: Ceratopogonidae) in Europe. Rev Sci Tech Int Off Epizoot. 2001 12;20(3):731–40. 11732415 10.20506/rst.20.3.1306

[45] Meiswinkel R. Discovery of a Culicoides imicola-free zone in South Africa: preliminary notes and potential significance. Onderstepoort J Vet Res. 1997 3;64(1):81–6. 9204508

[46] Purse BV, Tatem AJ, Caracappa S, Rogers DJ, Mellor PS, Baylis M, et al Modelling the distributions of Culicoides bluetongue virus vectors in Sicily in relation to satellite-derived climate variables. Med Vet Entomol. 2004;18(2):90–101. 15189233 10.1111/j.0269-283X.2004.00492.x

[47] Conte A, Goffredo M, Ippoliti C, Meiswinkel R. Influence of biotic and abiotic factors on the distribution and abundance of Culicoides imicola and the Obsoletus Complex in Italy. Vet Parasitol. 2007 12;150(4):333–44. 17997043 10.1016/j.vetpar.2007.09.021

[48] Calvete C, Estrada R, Miranda MA, Borras D, Calvo JH, Lucientes J. Modelling the distributions and spatial coincidence of bluetongue vectors Culicoides imicola and the Culicoides obsoletus group throughout the Iberian peninsula. Med Vet Entomol. 2008;22(2):124–34. 10.1111/j.1365-2915.2008.00728.x 18498611

[49] Purse BV, Falconer D, Sullivan MJ, Carpenter S, Mellor PS, Piertney SB, et al Impacts of climate, host and landscape factors on Culicoides species in Scotland. Med Vet Entomol. 2012 6;26(2):168–77. 10.1111/j.1365-2915.2011.00991.x 22103842

[50] Conte A, Ippoliti C, Savini L, Goffredo M, Meiswinkel R. Novel environmental factors influencing the distribution and abundance of Culicoides imicola and the Obsoletus Complex in Italy. Vet Ital. 2007 9;43(3):571–80. 20422536

